# SLEEP DISTURBANCES SHOW FEASIBILITY TO MONITOR DISABILITY PROGRESSION IN EARLY ALZHEIMER’S DISEASE

**DOI:** 10.1093/geroni/igad104.2759

**Published:** 2023-12-21

**Authors:** Jun Ha Chang, Ruiqian Wu, Ying Zhang, Matthew Rizzo, Jennifer Merickel

**Affiliations:** University of Nebraska Medical Center, Omaha, Nebraska, United States; University of Nebraska Medical Center, Omaha, Nebraska, United States; University of Nebraska Medical Center, Omaha, Nebraska, United States; University of Nebraska Medical Center, Omaha, Nebraska, United States; University of Nebraska Medical Center, Omaha, Nebraska, United States

## Abstract

Our objective is to assess the utility of real-world sleep to discriminate activities of daily living (ADL) decline in Alzheimer’s disease (AD). ADL decline warns of dementia and disability. Improved understanding of how sleep indexes ADL decline provides a platform for early monitoring and intervention to slow AD disability and progression. Fifty-seven impaired (mean age =76.7 years; mild cognitive impairment [MCI]: N=50, AD: N=7) and 30 unimpaired (mean age = 73.6 years) subjects participated. Demographics and the Dependence Scale (DS) were collected. The DS evaluated ADL decline from partner (e.g., spouse) report. The subject’s sleep was monitored for 3-months (N = 6,336 total days) using wrist-worn actigraphy and validated with sleep diaries. Sleep dysfunction was estimated using means and standard deviations (SD) of total sleep time, sleep latency, sleep efficiency, wakefulness after sleep onset (WASO), and awakening count. Logistic regression––adjusting for age, gender, and education––ranked the utility of sleep dysfunctions to discriminate ADL decline. Forty-six subjects (impaired: N=38, unimpaired: N=8) showed ADL decline (DS > 0). WASO variability (SD; b = 1.66, p < 0.001) and mean awakening count (b = -0.98, p = 0.003) best discriminated ADL decline. Sleep duration did not discriminate ADL decline. In line with prior literature, results underscore the role of sleep disturbances over duration in ADL decline and AD progression. Nightly awakenings show key clinical importance for real-world monitoring of AD-related disability risk. Further research may investigate the specificity and longitudinal reliability of sleep dysfunction to discriminate individual AD progression profiles.

